# Models of tendon development and injury

**DOI:** 10.1186/s42490-019-0029-5

**Published:** 2019-11-29

**Authors:** Sophia K. Theodossiou, Nathan R. Schiele

**Affiliations:** Biological Engineering, University of Idaho, 875 Perimeter Dr. MS 0904, Moscow, ID 83844 USA

**Keywords:** Tendon, Tissue engineering, Embryonic development, Injury, Engineered models, Computational models, Mechanical loading, Growth factors, Stem cells

## Abstract

Tendons link muscle to bone and transfer forces necessary for normal movement. Tendon injuries can be debilitating and their intrinsic healing potential is limited. These challenges have motivated the development of model systems to study the factors that regulate tendon formation and tendon injury. Recent advances in understanding of embryonic and postnatal tendon formation have inspired approaches that aimed to mimic key aspects of tendon development. Model systems have also been developed to explore factors that regulate tendon injury and healing. We highlight current model systems that explore developmentally inspired cellular, mechanical, and biochemical factors in tendon formation and tenogenic stem cell differentiation. Next, we discuss in vivo*,* in vitro*,* ex vivo*,* and computational models of tendon injury that examine how mechanical loading and biochemical factors contribute to tendon pathologies and healing. These tendon development and injury models show promise for identifying the factors guiding tendon formation and tendon pathologies, and will ultimately improve regenerative tissue engineering strategies and clinical outcomes.

## Background

Tendons transfer forces from muscle to bone and are essential for movement. Unfortunately, tendons are frequently injured [1], and their poor healing ability results in long-term loss of function [2]. Medical interventions, including surgical and non-surgical treatments, physical therapy, steroid injections, and anti-inflammatory medications have limited efficacy, and re-rupture is common [3]. These poor outcomes motivate the search for alternative treatment strategies aimed at preventing tendon injury, improving regenerative healing, and developing engineered tendon tissue replacements from stem cells. A major challenge for developing regenerative approaches has been a limited understanding of the factors that regulate tendon formation, injury, and healing.

Normal embryonic and postnatal tendon development are perfect models of tendon formation, but have been poorly understood. However, over the past 20 years, significant progress has been made in identifying underlying cellular, biochemical, and mechanical factors that regulate tendon formation during early development, and these important findings have been discussed in other recent reviews [4–17]. Using this new information, developmentally inspired approaches have recapitulated aspects of embryonic tendon cell differentiation and tendon formation in vitro. Here, we first focus on cell and explant tissue culture and tissue engineered model systems that have explored the cellular, biochemical and mechanical aspects of tendon development. In the second part of this review, we highlight model systems that may inform future clinical interventions for adult tendon injury. Specifically, we discuss in vivo, in vitro, and ex vivo models of tendon injury. In addition to experimental models, we highlight recent computational models that explore factors involved in tendon degeneration, injury, and healing.

## Main text

### Models of embryonic and postnatal tendon development

Tendon formation is initiated in early development as the musculoskeletal and connective tissues differentiate from embryonic mesoderm [15]. A few specific markers have been identified to distinguish tenogenesis (differentiation toward the tendon lineage) of progenitor and stem cells. Scleraxis, a transcription factor, is an early marker and regulator of tenogenesis [18–21]. Scleraxis regulates expression of tenomodulin, a late stage tenogenic marker [22, 23]. Mohawk is another transcription factor and regulator of tendon differentiation and formation [24]. The increased presence of collagen type (Col) I also indicates tenogenesis [25], but collagen production alone is not indicative of tenogenic differentiation since it is a major component of other musculoskeletal tissues such as bone and skin. However, the development of an aligned collagen structure and mechanical function can indicate appropriate tenogenesis and tendon formation. Taken together, this set of tenogenic markers (scleraxis, mohawk, tenomodulin, collagen content and organization, and mechanical properties) has led to advancements in understanding tendon development. To determine regulators of tenogenesis, in vitro and engineered model systems have been developed to incorporate the key cellular (cell organization and environment), biochemical (growth factors and extracellular matrix), and mechanical (tissue elastic modulus and dynamic loading) cues that are characteristic of developing tendons (Table 1).

**Table 1:** Summary of developmental tendon models

Developing Tendon Characteristics	Model Characteristics	Model Outcomes	References
High cell density and low collagen content (Ansorge 2011) [26] (Chaplin 1975) [27] (Richardson 2007) [28] (Schiele 2015) [29]	Self-assembled cellular fibers	Upregulated expression of scleraxis and tenomodulin with loading; potential for scaffold-free, cellular self-assembly for single tendon fibers	Mubyana 2018 [34] Schiele 2013 [35]
	Embryonic tendon cells in fibrin gels	Upregulated tendon genes and collagen synthesis; improved tendon formation in fibrin gel vs collagen gel models	Kalson 2010 [36] Kapacee 2010 [33] Yeung 2015 [37] Breidenbach 2015 [38]
Cell-cell junction proteins (Cadherin-11 & N-Cadherin) (Richardson 2007) [28]	Chick tendon explants, fibroblasts, and mouse MSCs	Possible regulators of early tendon tissue formation; N-cadherin and cadherin-11 levels decreased with tenogenic induction	Richardson 2007 [28] Schiele 2013 [35] Theodossiou 2019 [39]
TGFβ2&3 (Pryce 2009) [21] (Kuo 2008) [30]	Mouse embryonic tendon progenitor cells and fibroblasts, and MSCs	TGFβ2 increased scleraxis and tenomodulin expression	Pryce 2009 [21] Brown 2014 [40] Brown 2015 [41] Havis 2014 [42] Havis 2016 [43] Chien 2018 [44]
	Mouse MSCs	TGFβ2 increased scleraxis and tenomodulin production; decreased N-cadherin and cadherin−11 production	Theodossiou 2019 [39]
	Human BM-MNCs and MSCs in fibrin gels	TGFβ3 increased collagen fibril synthesis, and upregulated TGFβ3, Col I, and Smad2	Kapacee 2010 [33]
Scleraxis and mohawk (Schweitzer 2001) [18] (Liu 2015) [31] (Otabe 2015) [32] (Shukunami 2018) [33]	Scleraxis knockdown in equine embryonic stem cells, and fetal and adult tendon cells	Decreased expression of Col I, COMP, and Sox9, and reduced cell survival in embryonic stem cells and fetal tendon cells with scleraxis knockdown; adult tendon cells unaffected	Bavin 2017 [45]
	Scleraxis knockout in mice, and scleraxis knockdown in isolated rat tendon cells	Decreased or absent tenomodulin expression at P1 in scleraxis ^−^/^−^ mice; tenomodulin expression reduced to 17% of control by scleraxis knockdown in rat tendon cells	Shukunami 2018 [46]
	Mohawk knockout in rats via CRISPR/Cas9	Heterotropic mineralization of Achilles tendons and tendon hypoplasia in 3 and 4-week-old rats; increased expression of Col II, Runx2, Aggrecan, COMP, and osteopontin in patellar tendon cells	Suzuki 2016 [47]
	Overexpression of mohawk and scleraxis in mouse MSCs and cell sheets	Increased expression of Col I, biglycan, Col III, Col V, Col XIV, decorin, fibromodulin, tenascin C, tenomodulin, and scleraxis via binding to the TGFβ2 promoter	Liu 2015 [31]
	Overexpression of mohawk in human and mouse bone marrow-derived MSCs	Increased expression of Col I, tenomodulin, tenascin C, tenascin XB, scleraxis	Otabe 2015 [32]
	Overexpression of scleraxis in human MSCs in a silk-collagen scaffold	Increased expression of tenogenic genes, cell alignment, and fibril diameter	Chen 2014 [48]
FGF4 (Edom-Vovard 2002) [49] (Brent 2005) [50] (Havis 2014) [42]	Mouse MSCs and chick limb explants	Species-specific scleraxis expression: decreased in mouse or increased in chick	Havis 2014 [42] Havis 2016 [43]
	Mouse embryonic tendon progenitor cells and MSCs	No changes or decreased scleraxis expression	Brown 2014 [40] Brown 2015 [41]
BMPs (Lorda-Diez 2014) [38] (Liu 2015) [31] (Otabe 2015) [32]	Chick progenitor mesodermal cells	Transient gene expression determines response to BMP isoforms	Lorda Diez 2014 [54]
	Human bone marrow-derived MSCs	BMP-12 increased mohawk, scleraxis, Col I, tenascin XB, and decorin expression	Otabe 2015 [32]
LOX and Mechanical Stimuli (Marturano 2013) [51] (Pan 2018) [52]	Embryonic chick tendon and limb explants	Paralysis decreased elastic modulus and LOX, hypermotility increased LOX and elastic modulus, LOX inhibition decreased elastic modulus	Pan 2018 [55]
Lengthening/slow stretching (Hamburger & Hamilton 1951) [53]	Embryonic chick tendon cells in fibrin gels stretched 2 mm/day	Increased collagen fibril diameter, packing volume, and stiffness	Kalson 2011 [56]
Elastic modulus (Marturano 2013) [51]	RGD-functionalized alginate gels with embryonic-mimicking elastic modulus	Scleraxis, Col XII, and Col I gene expression regulated by elastic modulus	Marturano 2016 [57]
Progressive mineralization of tendon to bone attachment (Thomopoulos 2010) [12]	FEA model of cell- and tissue-level stress concentrations	Cell-level stresses much higher than tissue-level stresses; higher stresses may drive enthesis formation	Liu 2014 [58]

### Cellular cues

**Fig. 1:**
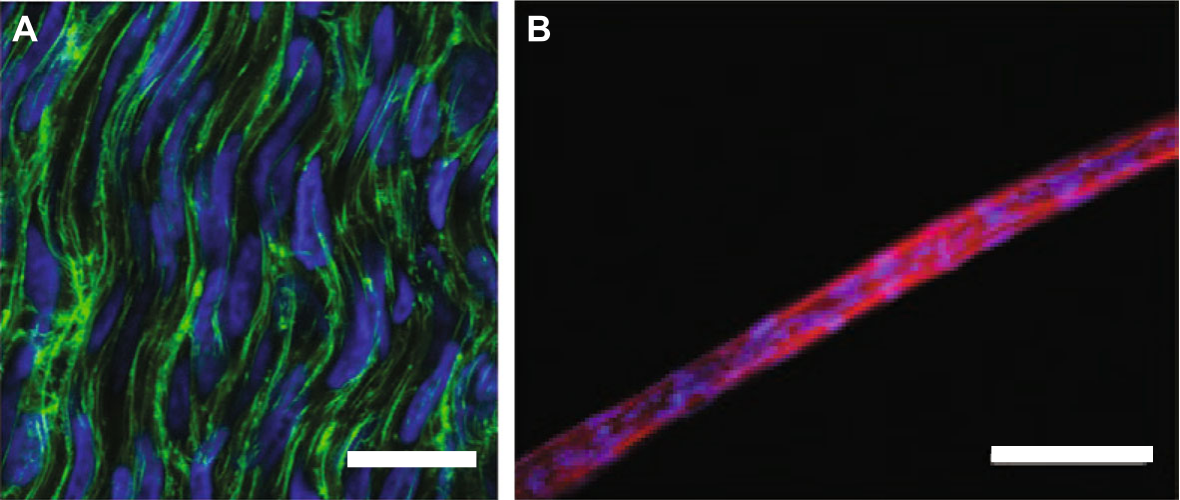
Embryonic tendon and a cellular fiber model. **a** E11 chick calcaneal tendons have high cell density and an organized actin cytoskeleton network. Actin cytoskeleton (green) and cell nuclei (blue) show actin filaments in embryonic tendon that appear to form a continuous network between adjacent cells. Scale bar = 10 μm. **b** A self-assembled cellular tendon fiber to mimic the high cell density of embryonic tendon, following 7 days of mechanical loading in vitro. Actin cytoskeleton (red) and cell nuclei (blue) show high cellularity, actin stress fiber organization and nuclear elongation. Scale bar = 100 μm. **a** reprinted with permission by Wiley Periodicals, Inc. from Schiele et al. 2015 [29]. **b** reprinted with permission by Mary Ann Liebert, Inc. from Mubyana & Corr 2018 [34]

Embryonic and early stage postnatal tendon is highly cellular and collagen content is relatively low, compared to adult tendon [26, 27, 29, 51, 59, 60]. For example, collagen content of Achilles tendons from postnatal day (P)4 mice is less than 3% of the dry weight [26], and in 1 week old sheep, cells account for nearly 33% of the tendon volume [59]. High cell density and cell organization in developing tendons may contribute to the organized and aligned collagen fibrils found in mature tendons. Based on scanning electron microscopy (SEM) imaging of embryonic tendon, it was proposed that embryonic tendon cell condensation and alignment of the cell’s plasma membrane channels, where collagen fibrils may be released into the extracellular space by the cells, regulate collagen fibril alignment [28]. The cell-cell junction protein cadherin-11 was demonstrated to play a role in embryonic tendon cell organization. When cadherin-11 was knocked down in isolated and cultured whole chick metatarsal tendons at embryonic day (E)13 using small interfering RNA (siRNA), the cells appeared to move apart, and plasma membrane channels and collagen fibrils were disrupted [28]. In a different study, serial block face-SEM was used to visualize cells in embryonic, neonatal, and postnatal mouse tail tendons [61]. Throughout development, the number of cells per unit volume decreased, but direct cell-cell contacts were maintained [61]. A study in E8 to 11 chick calcaneal tendons showed that the tendon progenitor cells formed an aligned and organized actin cytoskeleton network that appeared to be continuous between adjacent cells (Fig. 1a) [29]. Disrupting the actin cytoskeleton with blebbistatin in E10 calcaneal tendons decreased tendon elastic modulus. Similarly, the elastic modulus of embryonic tendon cell-seeded alginate gels decreased with blebbistatin treatment [29]. These findings suggest that the actin network of embryonic tendon cells contributes to the mechanical properties of the developing tendon. Taken together, these developmental studies underscore the role of tendon progenitor cells in tendon tissue formation, and suggest that their content and organization are important considerations in engineered models.

A few in vitro engineered model systems have been developed to mimic the high cell density of embryonic and neonatal tendons. A scaffold-free approach used directed cell self-assembly to recapitulate the high cell density and low collagen content associated with embryonic tendon [35]. 3-dimensional (3D) channels were laser micromachined into agarose gels, which were lined with a thin coating of fibronectin and seeded with neonatal fibroblasts. The channels directed cell self-assembly into single fibers with high cell density, and an organized and aligned cell structure [35]. Cells in the fibers contained cadherin-11, the cell-cell junction protein found in embryonic tendons [28]. In a different study, uniaxial cyclic tensile loading of the cellular fibers for 1, 3, and 7 days improved tendon fiber formation [34]. The fibroblasts forming the fibers had aligned and elongated cell nuclei and actin filaments (Fig. 1b). Scleraxis and tenomodulin gene expression increased in loaded fibers on day 1, and tenomodulin increased between day 1 and 7. Interestingly, none of the unloaded control fibers survived past day 3 [34]. In this model, loading appeared to counteract the self-generated static tension that arises in the cellular fibers. It is possible that only the loaded cellular fibers had established enough structure to support long-term fiber formation. These cell-based, scaffold-free models offer the advantage of combining high cell density with mechanical stimulation, making them a useful system for investigating key cellular aspects of early tendon development in a controlled in vitro environment.

Fibrin gels have also been used as in vitro model systems to explore what roles cells may be playing in embryonic tendon formation. Cell encapsulated in fibrin gels, formed from thrombin and fibrinogen crosslinking, can mimic the soft, 3D structure, and high cell density representative of embryonic tissues, without introducing exogenous collagen matrix. E13 chick metatarsal tendon cells seeded into fibrin gels at ~ 1.5 million cells/mL and cultured for up to 42 days resulted in tissue constructs that appeared similar to embryonic tendon, with newly synthesized collagen fibrils aligned along the axis of tension [36]. This embryonic-mimicking model system was then used to explore how contraction by the embryonic tendon cells may regulate mechanical development. When actin cytoskeleton-mediated cell contractility was disrupted for 24 h using cytochalasin D and blebbistatin, the mechanical properties of the tissue constructs failed to increase, even though collagen production was not altered [36]. This model implies that development of tissue mechanical properties may depend on contractility of the embryonic tendon cells. Taken together, engineered models have revealed the contributions of cell contractility, the actin cytoskeleton, and cell-cell junctions to tendon formation. However, the mechanisms by which cells regulate tendon development remain an ongoing area of study. Alongside these cell-level contributions, biochemical and mechanical cues may also guide tenogenesis.

### Growth factors and biochemical factors

A number of growth factors have been identified in embryonic tendon development, but transforming growth factor beta (TGFβ) has emerged as a critical tenogenic regulator. TGFβs and their receptors (TGFβR1 and TGFβR2) have been found in embryonic chick [62] and mouse [21] tendon. Chick calcaneal tendons from E13 to 16 were evaluated for TGFβ1, 2, 3, TGFβR1 and TGFβR2 using immunohistochemistry [62]. TGFβ2 and 3, and TGFβ receptors were detected at all ages in the tendon midsubstance, but TGFβ1 was not observed. In embryonic mice, TGFβs were found to regulate scleraxis expression and tendon formation [21]. No tendons formed in the limbs, trunk, tail, and head of TGFβ2 and TGFβ3 double knockout mice at E14.5, even though tendon progenitor cells were present, indicating that TGFβ signaling is required for maintenance of the tendon phenotype [21]. Taken together, TGFβs are critical to embryonic tendon formation in vivo.

Based on these findings in developing embryos, a number of studies have explored TGFβs in developmental and tissue engineered in vitro models. Mouse embryonic fibroblasts and mouse mesenchymal stem cells (MSCs) (C3H10T1/2 cells) both increased scleraxis expression when treated with TGFβ2 in culture [21]. In another study, mouse tendon progenitor cells, isolated from the limbs and axial skeleton at different ages (E13 to 17, and P7), were treated with either TGFβ2, cyclic tensile loading (1% strain, 0.5 Hz), or fibroblast growth factor (FGF)4, a member of the FGF/ERK/MAPK signaling pathway [40]. TGFβ2 treatment enhanced scleraxis gene expression across all ages in both axial and limb tendon progenitor cells. When E16.5 tendon progenitor cells were treated with combinations of TGFβ2, FGF4, and cyclic loading, scleraxis gene expression was upregulated in all treatment groups that included TGFβ2 [40]. In a similar study, E14 mouse tendon progenitor cells were compared directly to adult mouse bone marrow-derived MSCs [41]. MSCs had increased scleraxis gene expression with TGFβ2 treatment alone, and when TGFβ2 was combined with loading. FGF4 treatment alone decreased scleraxis [41], even though FGF4 had been identified in early stage embryonic mouse and chick tendon development [49, 50]. As before, scleraxis gene expression by embryonic tendon progenitor cells was upregulated in all treatment groups that included TGFβ2 [41].

To further assess the ability of TGFβ2 and FGF4 to drive tenogenesis, E3–4 chick forelimbs were grafted with beads containing FGF4, TGFβ2, FGF4 with a Smad2/3 inhibitor (SIS3), or TGFβ2 with a FGF/ERK/MAPK inhibitor (PD184352) [43]. Both FGF4 and TGFβ2 treatment increased scleraxis expression, and the Smad 2/3 and FGF/ERK/MAPK pathways regulated tenogenesis independently, as neither inhibitor downregulated scleraxis expression. When evaluated in chick and mouse limb explant cultures, FGF4 upregulated scleraxis expression in chick limbs, but downregulated scleraxis in E9.5 mouse limbs. TGFβ2 upregulated scleraxis in both animal models [43]. With scleraxis, tenomodulin was also upregulated in chick forelimb explants from E6.5 and 7.5 treated with TGFβ2 and FGF4. Additionally, E5.5, 6.5, and 7.5 chick limbs paralyzed during explant culture using decamethonium bromide (rigid paralysis) and pancuronium bromide (flaccid paralysis) had downregulated expression of scleraxis and tenomodulin [43]. FGF4 restored scleraxis expression in paralyzed chick limbs [43]. FGF4 was not tenogenic for mouse limb cells, where it inhibited scleraxis expression [43], in agreement with other in vitro models [40]. In the absence of FGF4, TGFβ2 was sufficient to maintain scleraxis and tenomodulin expression in immobilized chick limbs [43]. Taken together, these studies suggest variations in TGFβ and FGF signaling during embryonic tendon development between species, with only TGFβ2 able to induce tenogenesis in both mouse and chick. These results also indicate that TGFβ2 and FGF4 signaling may be initiated by mechanical stimuli from muscle contractions, to induce and maintain tenogenesis.

TGFβ2 was also used to explore tenogenic differentiation in mouse MSCs [42]. TGFβ2 treatment upregulated tenogenic genes via the Smad2/3 pathway, as a Smad 2/3 inhibitor (SIS3) eliminated TGFβ2-induced scleraxis expression [42]. In the same study, chemically blocking TGFβ receptors prevented tenogenic gene upregulation. A transcriptomic analysis of developing E11.5 to 14 tendons showed upregulation of several FGF ligands during differentiation, but downregulation of MAPK signaling [42]. The role of FGF signaling was then assessed in mouse limb explants [42]. A FGF/ERK/MAPK inhibitor (PD184352) activated scleraxis expression in explants from E9.5 or later, while activation of the FGF pathway downregulated scleraxis, consistent with prior studies [40]. Taken together, the results of these in vitro mouse and chick models suggest multiple growth factor-mediated pathways through which tendon development is initiated, modulated, and maintained, but highlight the pro-tenogenic impacts of TGFβ2.

Genetically manipulated cells have been utilized in other in vitro models of tendon development to investigate the role of Smad signaling in TGFβ2-induced tenogenesis. In addition to Smad2/3, TGFβ may drive differentiation of tendon and cartilage through Smad4 [63]. To explore Smad4 in tenogenesis, 3D fibrin gels were seeded with mouse embryonic fibroblasts modified by adenovirus-Cre-mediated floxing to knockout Smad4 [44]. Smad4 knockout cells still showed enhanced tenogenic differentiation with TGFβ2 treatment, but without TGFβ2-induced proliferation [44], suggesting that regulators of tendon cell proliferation are important to consider. While scleraxis expression remained higher in Smad4 knockout cells treated with TGFβ2 than wild type controls, untreated Smad4 knockout cells stained more strongly for glycosaminoglycans (GAGs), suggesting potential chondrogenic differentiation [44]. This in vitro developmental model demonstrated the role of TGFβ2 and Smad4 in regulating tenogenesis.

Tenogenic induction via TGFβ2 was also explored in mouse MSCs over 21 days in vitro. TGFβ2 treated cells showed fibroblastic morphology and enhanced proliferation, while protein levels of scleraxis increased at day 14 and 21, and tenomodulin increased at day 21 [39]. Cell-cell junction protein levels of N-cadherin and cadherin-11 decreased at all timepoints, and connexin 43 increased before trending downwards [39]. This study further showed that TGFβ2 may be useful in tenogenic induction of MSCs, and that cell-cell junctions found in embryonic tendon (cadherin-11, N-cadherin, and connexin-43) [28, 64], may also be regulated during tenogenesis.

While TGFβ2 appears to regulate tenogenesis, recent work has focused on identifying regulators of TGFβ2. Mohawk was found to bind to the TGFβ2 promoter, indicating mohawk directly influences TGFβ2 gene expression [31]. Overexpression of mohawk in cell sheets cultured from mouse MSCs increased gene expression of scleraxis, tenomodulin, biglycan, decorin, fibromodulin, tenascin C, and Col I, III, V, and XIV [31]. Ectopic expression of mohawk and scleraxis both individually decreased the osteogenic and adipogenic potential, as well as the self-renewal capacity of MSCs, while neither transcription factor affected the chondrogenic capacity of the cells [31]. Finally, mohawk was found to more efficiently promote tenogenesis compared to scleraxis ectopic expression, as ectopic mohawk expression resulted in a higher upregulation of fibromodulin, tenomodulin, and Col I, III, and V, as well as larger Col I fibril diameters within the cell sheets [31]. In a different study, mohawk overexpression in human bone marrow-derived MSCs upregulated expression of tenomodulin, tenascin C, tenascin XB, and Col I after 7 days, compared to controls [32]. Early growth response (EGR) 1, a recently identified tenogenic transcription factor [25], has also been explored as a potential regulator of TGFβ2. However, despite evidence that mohawk directly drives TGFβ2 expression [31], overexpression of both mohawk and scleraxis in vitro failed to increase expression of EGR1 and 2 [32], indicating another mechanism may be responsible for TGFβ2 regulation via EGRs. Collectively, mohawk appears to influence tenogenesis alongside scleraxis, and acts via TGFβ2 signaling, though additional studies are needed to determine how TGFβ2 is regulated during tendon development.

The role of mohawk in tenogenic differentiation was further demonstrated in vivo*.* Mohawk knockout rats generated via CRISPR/Cas 9 gene editing showed heterotopic ossification of the Achilles tendon at birth, and at 3 and 4 weeks of age [47]. This is an interesting finding, especially considering that heterotopic mineralization is frequently observed in human tendinopathies [65]. Furthermore, mohawk knockout rats had systemic hypoplasia of tendons, similar to mohawk knockout mice [24]. Cells derived from the patellar tendons of 3 week old Mohawk knockout rats had upregulation of chondrogenic and osteogenic genes, compared to cells from 3 week old mohawk ^+^/^+^ rats [47]. In the same study, overexpression of mohawk via retroviral transduction of patellar tendon-derived cells from the knockout rats suppressed chondrogenic, osteogenic, and adipogenic differentiation, consistent with similar findings in mouse.

Overexpression and knockdown of scleraxis have also been used to explore tenogenesis. Overexpression of scleraxis in human embryonic stem cell-derived MSCs seeded onto knitted silk-collagen scaffolds increased tenogenic gene expression, cell alignment, and collagen fibril diameter, compared to control cells [48]. Disruption of scleraxis negatively impacts tenogenesis. Scleraxis-null mice have tendon hypoplasia, complete loss of some tendons, and diminished tenomodulin expression [18, 20, 46]. Tendon cells isolated from P7 and P14 rats and treated with siRNA to knockdown scleraxis had tenomodulin expression that was reduced to 17% of the expression levels in control cells [46]. Another study knocked down scleraxis expression in equine embryonic stem cells, and adult and fetal tendon cells [45]. Scleraxis knockdown in fetal tendon cells significantly reduced Col I, cartilage oligomeric matrix protein (COMP) and Sox9 (a cartilage marker) gene expression, and reduced cell survival and tissue formation in 3D culture [45]. Interestingly, adult tendon cells were not affected by scleraxis knockdown. Overall, scleraxis appears necessary to maintain tenogenic differentiation, possibly through regulation of tenomodulin. Since tenogenesis is mediated at least in part by TGFβ2, the relationship between scleraxis, mohawk, TGFβ2, and tenomodulin requires further investigation.

TGFβ3 treatment and cell type were explored in an embryonic-like tendon formation model in vitro using human bone marrow-derived MSCs and bone marrow-derived mononuclear cells (BM-MNCs) [33]. Only MSCs produced embryonic tendon-mimicking collagen fibrils and fibropositors (cell structures that assemble fibrils) when cultured in fibrin gels under static tension for 7 days. TGFβ3, Col I, and Smad2 were upregulated in MSCs, and MSC contractility was prevented when treated with a Smad2 inhibitor (SB431542). TGFβ3 treatment increased collagen fibril synthesis, and upregulated TGFβ3, Col I, and Smad2 in MSCs and BM-MNCs, illustrating a potential role for TGFβ3 in augmenting the tenogenic potential of human stem cells. In other studies, TGFβ3 is chondrogenic [66], but these results suggest the tenogenic or chondrogenic effects of TGFβ3 may depend on factors such as cell type, tension generated by cell contractility, or characteristics of the engineered matrix.

Other growth factors have been explored in model systems of tendon development in vitro. Bone morphogenetic proteins (BMPs), members of the TGFβ family, are involved in musculoskeletal tissue and tendon development [67, 68–71] and induce tenogenic differentiation. Human bone marrow-derived MSCs treated for 5 days with BMP-12 increased expression of mohawk, scleraxis, Col I, tenascin XB, and decorin, compared to control cells, but tenomodulin levels were not impacted [32]. BMP-12 was also found to increase tenogenic gene expression in adipose-derived [72] and bone marrow-derived [32, 73] stem cells, making BMP-12 useful for inducing tenogenesis across multiple cell lines.

The role of other BMP isoforms in limb development was examined via in situ hybridization of chick limb autopods from E6.5 and E8, and in vitro micromass culture of E4.5 chick progenitor mesodermal cells isolated from limb buds [54]. Cells in micromass culture were treated throughout 12 days with exogenous BMP-2, 4, 5, and 7, as well as growth and differentiation factor (GDF)-5. In 2-day cell cultures treated with BMP-2 for 6 h, and in 4-day cell cultures treated with BMP-2 for 6 h, scleraxis expression was downregulated. Inhibition of BMP-2 upregulated scleraxis in 2-day cultures, but surprisingly, scleraxis was downregulated in 4-day cultures treated with a BMP inhibitor (AB204). These findings indicate that the cellular response to available BMPs depends on transient gene expression occurring in the target cells at the time of BMP signaling, and can vary based on culture day [54]. Understanding the variable cell responses to the same signaling pathway during differentiation provides new opportunities for understanding the spatiotemporal regulation of tenogenesis.

In addition to growth factors, several in vitro models have examined potential biochemical contributions of the extracellular matrix (ECM) during tenogenesis [74]. When E14 chick metatarsal tendon cells were cultured in fibrin or collagen gels, the gene expression profiles of cells in fibrin were most similar to native embryonic tendons, whereas cells in collagen gels had expression profiles more similar to cells in 2D culture, with an overall reduction in mechanotransduction-associated gene expression [37]. In addition to an ellipsoid cell morphology and parallel alignment, cells in fibrin constructs secreted their own de novo collagen matrix, which occurs in normal development [37]. Similarly, tendon and ligament progenitor cells from E17.5 scleraxis-GFP mice displayed increased collagen alignment and linear region elastic modulus when seeded in fibrin gels, compared to collagen gels. Cells in fibrin gels also had increased scleraxis, tenascin C, and fibromodulin expression after 14 days in culture [38]. Based on these studies, embryonic tendon may be better represented by in vitro models that incorporate minimal collagen matrix, which mimics the low collagen content found in developing tendons [26, 51].

Embryonic tendon cells produce matrix metalloproteinases (MMPs), enzymes that can degrade collagen and other proteins that may regulate the cell’s local biochemical environment. MMP-2, membrane type (MT)1-MMP, and MT3-MMP are present within tendon during embryonic development [75–77], and these MMPs may play a role in tendon tissue formation. Based on its presence in embryonic tendon, MT1-MMP was explored in an in vivo rat rotator cuff injury model [78]. Fibrin glue seeded with bone marrow-derived MSCs genetically manipulated to overexpress MT1-MMP was injected into a supraspinatus tendon injury. Tendons repaired with MT1-MMP overexpressing MSCs had improved mechanical properties and more fibrocartilage at 4 weeks post-injury, compared to control MSCs, suggesting that MT1-MMP augmented the healing process [78]. Based on these findings, MMPs deserve further study in models of tendon formation.

Overall, model systems have applied growth factors and biochemical cues identified in embryonic tendon development to influence tendon formation in vitro. TGFβ2 has been increasingly explored, as it appears to induce tenogenesis across a range of in vitro systems. Future model systems need to identify how TGFβ2 is produced and controlled to direct tendon formation. Interactions between biochemical cues (ECM and growth factors) are complex and may vary based on the cell type and species used, the timing and concentration of each biochemical cue, and the presence of mechanical loading. Such interactions need to be further explored in isolation and combination.

### Mechanical factors

#### Elastic modulus

Elastic modulus, the measure of a material’s resistance to elastic (i.e., non-permanent) deformation, is a factor that may guide stem cell differentiation [79, 80], and a few studies have measured the elastic moduli of embryonic tendons. Tensile testing showed that elastic moduli of E13 to 18 chick tendons range from approximately 200 kPa to over 20 MPa [36, 81, 56]. Nanoscale and microscale elastic moduli of chick calcaneal tendons from E5.5 to 17, measured by force volume-atomic force microscopy, increase nonlinearly from 7 to 21 kPa, and from 5 to 108 kPa, respectively [51]. These increases in elastic modulus occur simultaneously with differentiation of tendon progenitor cells, and may be an important tenogenic factor that several model systems have explored.

To identify the impact of elastic modulus on tenogenesis, alginate hydrogels were designed to mimic the elastic modulus of embryonic tendon at specific developmental stages [57]. Alginate hydrogels functionalized with arginyl-glycyl-aspartic acid (RGD), to enable cell attachment, were tuned using a combination of alginate concentration and calcium crosslinking density to have nanoscale elastic moduli from 3.4 to 20.1 kPa, representing the nanoscale elastic moduli of embryonic chick tendon from prior to E5.5 and up to E17 [57]. Tendon progenitor cells isolated from E11 chick calcaneal tendons were encapsulated in the 3D alginate hydrogels and cultured for 7 days in vitro. Scleraxis and Col XII gene expression increased at the highest elastic modulus (representing late stage embryonic tendon). Col I expression was downregulated at elastic moduli representing middle and later embryonic stages, whereas tenomodulin and Col III were not affected by elastic modulus [57]. This model suggests that embryonic tendon mechanical properties impact tenogenic markers, but additional factors may be needed, as late stage tendon markers (tenomodulin) were not affected. It is also possible that embryonic magnitudes of elastic moduli are not fully representative of the tenogenic environment. Tendon formation continues throughout postnatal development with increases in differentiation markers [82], collagen content, and mechanical properties [26, 59]. For example, linear region elastic modulus of postnatal mouse Achilles tendon increases from approximately 87 MPa at P4 to 544 MPa at P28, and toe region elastic modulus increases from 25 MPa to 72 MPa [26]. Elastic modulus of postnatal tendon can serve as a template for models aiming to mimic the complete developing tendon environment. As the stress-strain relationship in tendon is non-linear [83], the elastic modulus (e.g., toe region or linear) that impacts tenogenesis needs to be explored. Furthermore, tendon material properties can be evaluated at nano- and microscales (e.g., atomic force microscopy) or bulk scale (e.g., uniaxial tensile test), but how each scale impacts cells is unknown and challenging to uncouple. Model systems exploring the effects of bulk and cell-level material properties on tenogenesis are needed.

#### Static and dynamic tensile loading

Mechanical loading is a critical factor in tendon development, and has been highlighted in recent reviews [4, 15, 17]. In the developing embryo, quasi-static or static loading may result from limb lengthening or the contractile forces generated by the tendon cells themselves, while dynamic loading results from skeletal muscle contractions. In vitro bioreactor systems have been developed to apply mechanical stimuli [44, 84–86], with loading enhancing tenogenic markers [87–89], collagen production [30], and mechanical properties [36, 90–92] of engineered tissues. Here, we discuss developmentally mimicking tendon models that investigate the effects of static and dynamic loading.

Tendon cells isolated from adult human semitendinosus and gracilis tendons and cultured in fibrin gels under self-generated static tension produced embryonic-like tendon tissue, with increased collagen fibrillogenesis and deposition of aligned collagen fibrils [30]. After 10 days of culture, force-displacement curves displayed the characteristic toe and linear regions of tendon [30]. The cells produced Col I, III, XII, and XIV, fibronectin, integrin α5, and small-diameter collagen fibrils and fibropositors, all components found in embryonic tendon [30]. With the right environment and self-generated static tension, adult tendon cells may behave as embryonic tendon cells, and develop an embryonic tendon-like tissue. However, in a different study, fibrin gel contraction by embryonic tendon cells occurred at a faster rate than adult tendon cells [36]. While adult tendon cells may form embryonic-like tissues in vitro, the ability for embryonic tendon cells to rapidly modify their microenvironment by contraction may result in functionally distinct tissues and should be considered when evaluating cell types for in vitro developmental models.

**Fig. 2:**
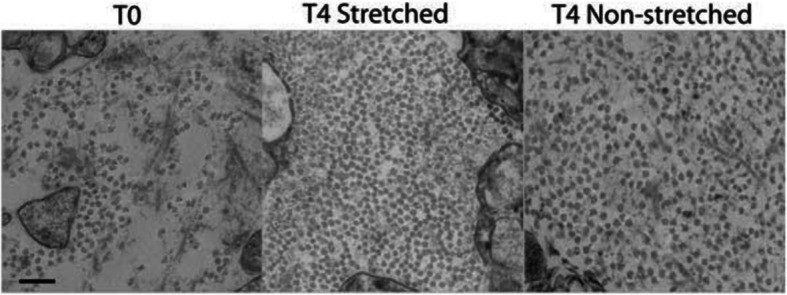
Stretch influences collagen fibril formation in an embryonic tendon model. Transmission electron microscopy images of fibrin gel tendon constructs seeded with embryonic chick metatarsal tendon cells at day 0 (T0), and after 4 days (T4) with and without stretching. Slow stretching (2 mm/day) increased collagen organization and collagen fibril packing volume in this in vitro model of embryonic tendon formation. Scale bar = 250 nm. Figure reprinted with permission by Wiley Periodicals, Inc. from Kalson et al. 2011 [56]

Slow stretching has been explored in a model of tendon formation, based on the observed increase in limb length during development [93]. Specifically, lengthening of the third metatarsal in chick from E10 to 14 was proposed to stretch the developing metatarsal tendon. To mimic this, a slow continuous stretch was applied to embryonic chick metatarsal tendon cells seeded in fibrin gels [56]. Slow stretching (2 mm/day over 4 days to double the construct length from 8 to 16 mm) increased collagen fibril diameter, fibril packing volume, and stiffness, all characteristics of more mature tendon (Fig. 2) [56]. Unstretched controls resembled early stage embryonic tendon. Extrinsic stretch can be effectively applied to mimic in vivo stretch experienced by the developing tendon, but the appropriate magnitudes and timing for each tendon need further characterization.

Dynamic movement in the embryo is facilitated by developing muscles, whose concomitant development alongside tendons provides both mechanical and biochemical cues that drive tenogenic differentiation. Pax3 knockout mice (Pax3^Spd/Spd^), which lack skeletal muscle, show that tenogenesis is initiated even in the absence of normal myogenesis [94]. However, while initial tenogenic induction is independent of muscles, tendons are unable to elongate and are subsequently lost by E13.5 in Pax3^Spd/Spd^muscle-less mice [94], similar to prior studies in muscle-less chick limbs [49, 53, 95]. When muscles were intact, but genetically altered via a muscular dysgenesis (*mdg*) mutation to limit movement, tendon progenitors in the embryonic mouse forelimb were maintained at E12.5 [94]. However, tendons from *mdg* mice at E16.5 were smaller than in wild-type mice, though they were not diminished to the same extent as tendons from Pax3^Spd/Spd^muscle-less mice [94]. While muscles may not be required for tenogenic induction, several previous studies suggest that muscles and subsequent mechanical stimuli are needed for continued tendon development [49, 53, 55, 94, 95]. This was further demonstrated in a chick model. Chick embryos subjected to systemic rigid paralysis (using decamethonium bromide) for 48 h had reduced calcaneal tendon elastic modulus at E17, whereas hypermotility (using 4-aminopyridine) increased elastic modulus [55]. Lysyl oxidase (LOX), an enzyme involved in collagen crosslinking and embryonic tendon mechanical property development [96], was also assessed in calcaneal tendons in embryos and limb explant cultures from paralyzed and hypermotile chicks at E19 [55]. In embryos, paralysis reduced LOX activity, and when LOX was inhibited, hypermotility no longer increased elastic modulus. Overall, embryonic movements may regulate the formation of tendon mechanical properties through LOX-mediated collagen crosslinking. Based on these studies, in vitro models exploring mechanical loading may consider LOX-mediated mechanisms of tissue formation. Furthermore, exogenously applied LOX increased ultimate tensile strength and modulus in an engineered tissue model [97], suggesting that LOX can be successfully used to enhance tissue mechanical properties in vitro.

In vitro, cyclic loading representing contracting muscles during development has the potential to impact tenogenesis. For example, cyclic loading of mouse MSCs seeded in collagen gels increased scleraxis and Col I gene expression, over static controls [88]. Scleraxis also increased as a function of strain magnitude and number of loading repetitions. Similarly, cyclic loading enhanced tendon tissue formation and tendon gene expression in self-assembly models that captured embryonic tendon cellular cues [34, 35]. However, appropriate levels of loading (e.g., strain magnitude, frequency, rate, duration, etc.) for tendon formation are still unknown. The in vitro models reviewed here may be employed to determine these loading parameters in bioreactor systems isolated from other confounding factors associated with in vivo models. Determining the timing, intensity, and duration of tenogenic mechanical stimuli is a challenge for tendon tissue engineering, and will require additional in vivo and in vitro studies.

To explore mechanical loading parameters, computational models may be a good alterative, but have only been used for evaluating enthesis formation. The enthesis is a progressively mineralized fibrocartilage interfacial tissue that extends from the tendon to the bone insertion and is impacted by loading [11, 12, 52, 98]. A computational model of mineralization during enthesis formation was developed based on histological data from mice at P7, 10, 14, 28, and 56 [58]. The mineralization gradient was predicted to be driven by cell-level stress rather than tissue-level stress, which may allow for relatively small tissue-level stresses to drive mineralization via the larger effect exerted on individual cells [58]. Cell-level local stresses predicted by the model at early time points almost reached adult physiological levels, likely stimulating mineralization [58]. The development of this complex interface tissue has been explored in vivo [99, 100, 101]*,* but future engineered systems and computational models may be useful for understanding the mechanical and biochemical factors involved in enthesis and tendon formation.

### Models of adult tendon injury

Adult tendon contains a dense network of aligned and continuous collagen fibrils that are responsible for force transmission [102, 103]. Unfortunately, the incidence of tendon ruptures is increasing [1, 104, 105], and tendon heals as disorganized scar tissue that does not regain mechanical function [2, 106]. A major challenge has been a limited understanding of the numerous factors that influence tendon injury (e.g., tendinopathy and ruptures) and healing. Model systems and computational models have been developed to explore impacts of mechanical loading, biochemical factors, and inflammatory cytokines on adult tendon injury and healing (Table 2).

**Table 2:** Summary of tendon injury models

Injured Tendon Characteristics	Model Characteristics	Model Outcomes	References
Overuse injury	Downhill running in rats	Induced overuse injury in the supraspinatus	Soslowsky 2000 [107] Archaumbault 2006 [108]
	Bipedal downhill running in rats	Reduced stiffness and tensile strength; localized disintegration of collagen bundles	Ng 2011 [109]
	Uphill running in rats	Achilles tendons adapted to loading; no observable pathology	Heinemeier 2012 [110] Dirks 2013 [111]
Transection/Acute injury	Neonatal and adult mouse Achilles tendons	Regeneration observed in neonates, but not adults	Howell 2017 [112]
	Mouse supraspinatus tendons with full and partial transections	Different cell populations involved in healing of full versus partial injury; distinct cell lineages participate in healing response	Moser 2018 [113] Yoshida 2016 [114]
	Rat Achilles tendon partial transection repaired with scaffolds	Cells in scaffolds expressed mohawk during repair	Otabe 2015 [32]
	Mouse Achilles tendon full transections repaired with MSC sheets overexpressing mohawk	Mohawk-overexpressing MSC sheets resulted in increased collagen fibril diameter, visible crimp, increased stiffness, elastic modulus, maximum force and stress, and energy absorbed	Liu 2015 [31]
	Canine digital flexor tendons	Following injury, IL-1β upregulated 4000-fold, MMP-13 upregulated 24,000-fold	Manning 2014 [115]
IL-1β treatment	E15 and P7 mouse tendon cells treated with IL-1β	Higher expression of IL-6, TNFα, COX2, MMP-3 and MMP-13 in P7 compared to E15	Li 2019 [116]
	Human patellar tendon fibroblasts treated with IL-1β and strain	IL-1β and 8% strain upregulated MMP-1, COX2, and PGE2; IL-1β and 4% strain downregulated expression of MMP-1, COX2, and PGE2 compared to 8% strain	Yang 2005 [117]
	Adult and fetal equine tendon cells, and equine embryonic stem cells treated with IL-1β	Adult and fetal tendon cells upregulated MMP-1, −2, −3, −8, −9, and − 13, tenascin-C, Sox9, and downregulated scleraxis and COMP, compared to embryonic stem cells	McClellan 2019 [118]
Genetic knockouts	Tenomodulin knockout mice with transected and repaired Achilles tendons	Downregulation of Col I, tenascin-C, thrombospondin 2, and TGFβ1; upregulation of scleraxis, COMP, and proteoglycan 4	Lin 2017 [119]
	GDF-5 knockout mice subjected to Achilles tendon injury	Delayed healing and increased adipocytes in knockouts	Chhabra 2003 [120]
	Decorin-null and biglycan-null mice subjected to full thickness, partial width patellar tendon injury in adult and aged groups	Smaller diameter collagen fibrils, decreased cell density, and altered cell shape and collagen alignment in knockouts; biglycan influenced early healing, decorin influenced late healing	Dunkman 2014 [121] Dunkman 2014 [122]
Chronic Injury/Induced Tendinopathy	Transection or Botox-unloading of rat Achilles tendon	Irreversible loss of scleraxis expression with transection; partial loss and return of scleraxis with Botox	Maeda 2011 [123]
	Immediate or delayed repair of rat rotator cuff injury	Delayed repair had worse outcomes than immediate repair	Killian 2014 [124]
	TGFβ1 injection to rat Achilles	Warburg pathway, hypoxic, angiogenic, and glycolytic metabolism gene activation	Sikes 2018 [125]
	Collagenase injection in rat Achilles tendon	Increased IL-6 and MMP-9 in senescence-accelerated rats compared to senescence-resistant rats	Ueda 2019 [126]
	Carrageenan injection in rat patellar tendon; treatment with IL-1 receptor antagonist	Carrageenan decreased tendon length, and increased MMP activity and inflammation. Inflammation absent with IL-1 receptor antagonist	Berkoff 2016 [127]
Ex vivo Loading	Stress deprivation in rat tail tendons	Increased MMP-13 expression	Arnoczky 2007 [128]
	Stress deprivation in rat tail tendons	Stress deprivation decreased TIMP/MMP ratio; loading increased TIMP/MMP ratio	Gardner 2008 [129]
	Fatigue loading of rat flexor digitorum longus tendon loaded at low (6.0–7.0%), moderate (8.5–9.5%), and high (11.0–12.0%) tensile strain	Isolated fiber deformations at low strain; fiber dissociation and localized rupture, decreased stiffness, and increased hysteresis at high strain	Fung 2009 [130]
	Equine flexor and extensor tendon cells subjected to 10% biaxial cyclic loading	Collagen synthesis, proliferation, COMP expression as a function of tendon type	Goodman 2004 [131]
	Equine superficial digital flexor tendon fascicles cyclically loaded from 2–12% uniaxial strain and 1800 cycles	Increased expression of IL-6, COX2, C1, C2, and MMP-13	Thorpe 2015 [132]
	Bovine deep digital flexor tendons cyclically loaded from 1 to 10% strain	Collagen fiber disruption, kinks, and interfascicular network damage, and expression of IL-6, COX2, MMP-1, 3, and 13	Spiesz 2015 [48]
	Mouse patellar tendon cells isolated from 3-week old mohawk knockouts and subjected to 4% cyclic tensile loading	Increased chondrogenic gene expression (Col II, Aggrecan, COMP)	Suzuki 2016 [47]
Computational models	Cell- and tissue-level responses to strain simulated via Hill functions	Tissue-level response similar at low and high strain conditions	Mehdizadeh 2017 [133]
	Hill-type equations of human Achilles-soleus unit	Proteolytic damage leads to collagen fiber shortening; mechanical damage lengthens fibers	Young 2016 [134]
	Regression model of healing	Multiple differential predictors of early development and early developmental healing; however, no differential predictors of late development and late developmental healing	Ansorge 2012 [135]
	2D FEA simulation of “jumper’s knee” in Patellar tendon	Highest localized strain predicted successfully	Lavagnino 2008 [136]
	Agent-based model of collagen fibril alignment with applications in tendon loading during healing	Peak collagen alignment occurs at lower strain level than peak deposition; peak deposition occurs above damage threshhold	Richardson 2018 [137]
	Multiscale OpenSim model of cellular responses to various loading parameters	Single set of cellular response curves explained tendon behavior observed in several different experiments	Chen 2018 [138]
	Empirical model of patellar tendon response to aging and injury	Effects of aging and injury on patellar tendon mechanical properties predicted by damage models	Buckley 2013 [139]
	Empirical model of Achilles tendon response to decorin and biglycan knockout in aging mice	Model predicted changes in dynamic modulus resulting from decorin and biglycan knockout	Gordon 2015 [140]

#### Mechanical loading

##### Overuse injury

Adult tendon injury may be influenced by mechanical loading [128, 141–143]. Models to explore overuse injury have induced uphill and downhill treadmill running in animal models. Adult rats running on a 10° incline treadmill (1 h/day, 5 days/week) over 12 weeks had no observable Achilles tendon damage, compared to controls [110]. Elastic modulus and the ratio of failure stress to body weight increased in Achilles tendons from the running group. Running upregulated expression of Col III and insulin-like growth factor (IGF)-I, but downregulated TGFβ1, connective tissue growth factor (CTGF), and ECM components fibromodulin and biglycan, with no impact on Col I. Notably, these gene expression profiles are not observed in human tendinopathies [144]. The increased mechanical properties coupled with these changes suggest that tendons adapted to increased mechanical stimuli and exercise may maintain or improve tendon health, but this did not produce an overuse injury model [110]. These results were consistent with a study that found no histological evidence of tendon injury with uphill running in rats [111]. In contrast, downhill running on a 10° decline (17 m/min, 1 h/day, 5 days/week) for 4, 8, or 16 weeks induced an overuse injury in the supraspinatus tendon of the rotator cuff in adult rats [107]. Compared to unexercised controls, downhill running increased cellularity and rounded cell-shape, and decreased collagen fiber alignment, cross-sectional area, maximum stress, and elastic modulus [107]. In a follow-up study, 2 and 4 weeks of downhill running increased cartilage-associated gene expression for Col II, aggrecan, and Sox9 in the rat supraspinatus tendon, compared to nonrunning controls [108]. These rat models of tendon overuse demonstrate that some tendons can adapt to mechanical loading, while others display pathology, suggesting that specific tendons are more prone to overuse injuries, an important consideration for selecting an appropriate model system.

A potential limitation of rat models in overuse tendon injury is the difference in locomotion between bipedal humans and quadrupedal rats. To address this, a custom treadmill was used to allow adult rats to run downhill bipedally on a 20^o^ decline (1 h/day, 7 days/week) for 8 weeks. Achilles tendons of the running group had increased cell proliferation, a more ovoid cell morphology, and less organized ECM, with localized disintegration of collagen bundles. Bipedal running also reduced stiffness and ultimate tensile strength, compared to controls [109]. Achilles tendons did not appear to adapt to the increased loading demands with this magnitude of bipedal running, but appeared pathogenic, making this a potentially good model of Achilles tendon overuse injury. However, bipedal running in a normally quadrupedal animal may be a confounding factor. Model systems to mimic human adaption or overuse injuries in tendon are needed, and also must consider other potential factors including age, gender, systemic inflammation, co-morbidities, prior injuries, and lifestyle. Specific loading parameters such as duration and intensity also need to be explored, as studies in human Achilles tendon show adaption as a function of strain magnitude during loading [145, 146].

Ex vivo models have examined damage in tendons resulting from repetitive loading. Fatigue damage in isolated adult rat flexor digitorum longus tendons was assessed at low (6.0–7.0%), moderate (8.5–9.5%), and high (11.0–12.0%) peak levels of clamp-to-clamp tensile strain [130]. Samples were cycled between 1 and 16 N at 0.75 Hz until the desired strain magnitude was reached. Stiffness decreased and hysteresis increased, but only at high strain. Low strain led to isolated collagen fiber damage, but as strain increased, fiber dissociation and localized rupture were observed, and damaged fiber areas increased. This model expanded the range of strains that must be considered when assessing tendon damage, but used a relatively high strain magnitude (12%), which may account for the differences observed between strain magnitudes. Interleukin (IL)-1β, an inflammatory cytokine, and MMP-13 may also be impacted by strain magnitude applied to tendon [147]. Adult female rat patellar tendons were cyclically loaded in vivo between 1 and 35 N at 1 Hz until reaching 0.6% or 1.7% strain. Following 1 and 3 days of recovery, tendons elongated to 1.7% displayed microstructural damage and upregulated expression of MMP-13 and IL-1β, compared to the 0.6% group, which downregulated expression of both MMP-13 and IL-1β [147].

**Fig. 3:**
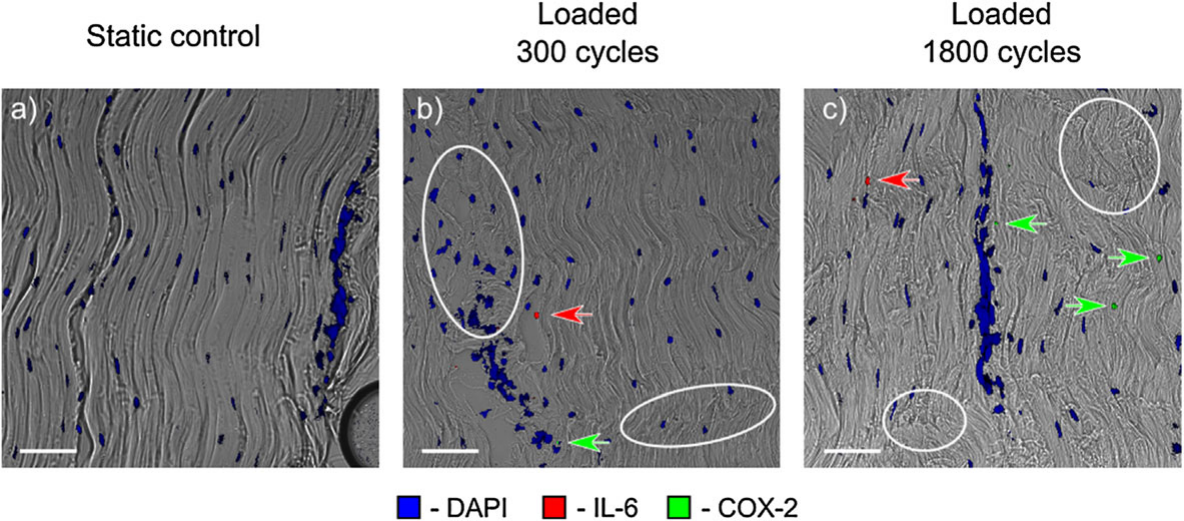
Mechanical loading impacts ex vivo tendon damage. Ex vivo static (**a**) and cyclically loaded (**b**, **c**) bovine flexor tendon fascicles immunostained for inflammatory markers IL-6 (red) and COX-2 (green), and co-labeled for cell nuclei (DAPI, blue). Fascicles and the interfascicular matrix of the loaded samples show damage (white ellipses), with collagen fiber kinks and interfascicular matrix disruption. IL-6 and COX-2 are found in loaded samples only, with COX-2 expression increasing with cycle number. Scale bar = 10 μm. Figure reprinted under a Creative Commons Attribution License from Spiesz et al. 2015 [147]

Other ex vivo models applied mechanical loading to isolated tendons and tendon fascicles. Equine superficial digital flexor tendon fascicles cyclically loaded from 2 to 12% uniaxial strain for 1800 cycles had increased levels of inflammatory mediators, IL-6 and cyclooxygenase 2 (COX2) [132]. Collagen degradation markers, C1 and C2, and MMP-13 activity were also increased, and cells appeared rounder and less elongated. Although these markers of tendon damage were increased, overall levels were relatively low, indicating a possible low-level inflammatory response. Low-level inflammation with loading may have implications for long-term tissue health, rather than inducing an acute injury. Similar results were obtained when bovine flexor tendons were cyclically loaded from 1 to 10% strain [148]. Loaded tendons had collagen fiber disruption and kinks, and interfascicular network damage, as well as expression of IL-6 and COX2, which were absent from non-loaded controls (Fig. 3). MMP-1, 3 and 13 were detected in interfascicular regions of loaded tendons, but only minimally detected in controls [148]. The interfascicular tissue involvement in the loading response is a novel finding of this model, and highlights a possible role in tendon pathology.

In vitro cell culture models have assessed effects of cyclic strain and growth factors on tendon cell behavior, as a function of tendon type. Equine tendon cells isolated from flexor and extensor tendons of fetal, P11, 8 month, and 4, 8, and 10 year old horses were cyclically loaded to 10% strain for 24 h, and treated with TGFβ1 or TGFβ3 [131]. TGFβ1, TGFβ3, and cyclic strain did not increase flexor tendon cell proliferation. Extensor tendon cell proliferation was increased by loading, but not by TGFβ1 or TGFβ3 treatment. TGFβ1 and TGFβ3 increased Col I and III production, incorporation of 3-hydroxyproline into the collagen, and COMP in both cell types regardless of whether cells were loaded, but when TGFβ1 or TGFβ3 were combined with loading, neither cell type had increased proliferation at any age. COMP and Col I and III synthesis was higher in flexor tendon cells from horses up to 8 months old, compared to flexor cells isolated from older horses. Interestingly, age had no effect on activity of extensor tendon cells. Tendon-specific responses to mechanical stimulation and aging emphasize the importance of controlling for tendon type in model systems.

In a different cell culture model, adult rat patellar tendon cells were loaded in vitro via hydrostatic pressure to 2.5 and 7.5 MPa [147]. Both loaded groups upregulated IL-1β and MMP-13 expression, compared to unloaded controls. siRNA knockdown of IL-1β partially suppressed loading-inducedMMP-13 expression and activity [147]. MMP-13 has been associated with human tendinopathies [149], and this model shows that MMP-13 expression may be regulated by loading and resulting inflammatory cytokines. Using model systems, loading parameters to induce an adaptive rather than pathogenic response may be identified and provide opportunities for clinical interventions incorporating loading.

While tendon over-loading may induce damage, under-stimulation also leads to pathology [150–152, 128, 153]. MMPs have been explored as mediators of load-dependent tendinopathy in ex vivo models of stress deprivation. Adult rat tail tendons subjected to 1 week of stress deprivation ex vivo increased MMP-13 gene expression and enzymatic activity, and inhibiting MMPs improved ultimate stress, tensile modulus, and strain at ultimate stress [154]. Mechanical loading also stimulates tissue inhibitors of metalloproteinases (TIMPs), which inhibit MMPs [129]. *S*tress deprivation of rat tail tendons ex vivo decreased the TIMP-1 to MMP-13 ratio, compared to cyclically loaded controls [129]. When tail tendons were subjected to 1, 3%, or 6% cyclic strain for 24 h, all groups increased the TIMP-1 to MMP-13 ratio [129]. Mechanically activating TIMPs may prevent MMP-mediated degradation. Mechanical stretch may also protect collagen fibers aligned along the axis of loading by hiding MMP-cleavable degradation sites within the collagen [155, 156–160]. Based on these models, stress deprivation in tendon may stimulate MMP production, while also making collagen more susceptible to MMP degradation, but these compounded effects need further study in vivo.

A few injury models have explored how mechanical loading impacts tendon healing. When a supraspinatus injury was cast immobilized in adult rats, mechanical, compositional, and structural properties improved, compared to injured groups allowed cage activity or allowed to run at 10 m/min for 1 h/day and 5 days/week [161]. Immobilized groups had upregulated chondrogenic genes, while exercise upregulated tenogenic genes [161]. Another study investigated Achilles tendon injuries in mice [162]. Healing of a bilateral full thickness, partial width excisional injury was evaluated at 0, 1, 3, or 6 weeks. A fatigue test showed initial decreases in tangent stiffness, dynamic modulus, and hysteresis immediately following injury that were not improved after 6 weeks of healing [163]. In a follow-up study, the hindlimbs were cast-immobilized in plantarflexion for 1 or 3 weeks following Achilles tendon transection, and then assessed after 16 weeks [163]. Tendons immobilized for 1 week had lower joint stiffness in plantarflexion than tendons immobilized for 3 weeks, though both were increased compared to transected controls with normal cage activity. Stride width during walking, tendon cross-sectional area, and laxity (the tendency of the tendons to elongate under fatigue loading) increased in mice immobilized for both 1 and 3 weeks, compared to uninjured controls. Secant stiffness remained at pre-injury levels, and tissues appeared histologically normal for both injured groups [163]. This model recreated immobilization periods consistent with conservative management of acute tendon injuries in humans, and showed some improvement in tendon mechanical properties. However, laxity may lead to joint dysfunction, and may be regulated by contractile tendon cells [164, 165], suggesting immobilization during healing did not return tendons cells to their normal function. Taken together, these injury models show that the mechanical environment may play a role in tendon healing.

##### Surgical injury models of the rotator cuff

Surgical models to induce injury have shown promise for identifying factors that influence rotator cuff healing. Partial and full detachment tears of the supraspinatus tendons in adult mice were induced by either insertion of a 26G needle through the central portion of the supraspinatus tendon into the insertion site at the enthesis, or a full transection and surgical repair using sutures [113]. Both injury models healed via scar formation, but the amount of scarring following full detachment and repair led to permanent impairments in gait and disruption of the architecture and organization of the enthesis. In the partial tear model, gait was not affected, but there was still considerable hypercellular scarring and increased cell density within the healing enthesis. In the same model, lineage tracing showed minimal scleraxis or Sox9 expression in the scar, suggesting that the scar-forming cells were not predominantly derived from tendon, articular cartilage, or unmineralized enthesis [113]. Axin2-expressing cells (indicating resident stem cell lineage) were not found in the scar of the partial tear model, but were the majority of cells detected in the scar of the full tear. Sox9-expressing cells were detected in the articular cartilage of the humeral head, the unmineralized enthesis fibrocartilage, and near the insertion in both the full and partial tear models [113]. These results suggest that distinct cellular mechanisms may operate in response to partial or full tear injuries of the rotator cuff.

Another surgical model developed a full-thickness injury by detaching the central portion of the supraspinatus tendons of adult mice [114]. Healing was assessed at 1, 2, and 5 weeks post-surgery along with evaluating smooth muscle actin, proteoglycan-4, and aggrecan-expressing cells at the site of healing. Two weeks post-surgery, proteoglycan-4 expressing cells were found in midsubstance and in the paratenon on the bursal side of the supraspinatus, as well as in the articular cartilage of the humerus and joint capsule, while smooth muscle actin-expressing cells were localized to the paratenon, blood vessels, and periosteum [114]. Aggrecan-expressing cells were found in the articular cartilage of the humerus, the unmineralized fibrocartilage at the supraspinatus tendon enthesis, and in the fibrocartilage cells of the acromioclavicular joint, but were not found elsewhere in the midsubstance, myotendinous junction, or paratenon [114]. The distal stump of the injured tendon underwent minimal remodeling, as indicated by a lack of labeled cells, but cells from both the bursal and articular surfaces appeared to contribute to healing, a novel finding in rotator cuff injury models [114]. Together these models have implications for the type of surgical model used to investigate rotator cuff injuries (i.e. partial or full transection). The identification of multiple distinct cell lineages participating in the healing process is interesting and worth exploring in chronic models of rotator cuff injury.

### Biochemical factors

#### Inflammatory cytokines

The inflammatory cytokines IL-6 and IL-1β have been implicated in tendinopathies [166, 167]. IL-6 and MMP-9 were upregulated in adult senescence-accelerated and senescence-resistant mice in response to collagenase type I injections in the Achilles tendon, compared to controls injected with saline [126]. Upregulation of IL-6 was higher in the senescence-accelerated mice compared to the senescence-resistant mice, suggesting the inflammatory response increases with age. IL-6 was also upregulated in tendon cells from bovine extensor tendon fascicles cyclically loaded to 30 and 60% of failure strain [168]. Compared to unloaded controls and fascicles loaded to 60% of failure strain, fascicles loaded to 30% of failure strain increased IL-6 and Col I expression and had no structural damage. Together, these findings suggest that IL-6 is involved in an adaptive response to loading and may be influenced by aging, but additional studies are needed to distinguish adaptive and pathological functions of IL-6.

IL-1β is a potent mediator of inflammation and is associated with tendon injuries [115]. IL-1β was upregulated 4000-fold, 1 day after a laceration injury in canine forelimb flexor tendons, and remained elevated compared to uninjured controls for 9 days post-injury [115]. In vitro, human patellar tendon cells treated with IL-1β and cyclically loaded to 8% strain for 4 h upregulated expression of MMP-1, COX2, and prostaglandin (PGE)2, compared to cells treated with IL-1β and stretched to 4% strain [117]. When compared to unstretched controls, 4% strain and IL-1β downregulated expression of MMP-1, COX2, and PGE2, while 8% strain and IL-1β upregulated MMP-1, COX2, and PGE2 [117]. This in vitro model shows that mechanical stimulation and IL-1β may mediate markers of tendinopathy.

In vivo tendon injury models show that embryos and neonates retain greater regenerative capacity than adults [169, 170]. For example, an Achilles tendon transection in neonatal (P5) mice showed regenerative healing, with a return to pre-injury mechanical properties and gait, while adult mice healed with scar and diminished mechanical properties [112]. IL-1β has been explored in model systems aimed at understanding the inflammatory responses in adult and fetal tendon cells. IL-1β treatment of adult equine tendon cells increased expression of MMP-1, 2, 3, 8, 9, and 13, as well as tenascin-C and Sox9 (a chondrogenic marker), and decreased expression of scleraxis and COMP, compared to IL-1β treated equine fetal tendon cells and tendon cells derived from equine embryonic stem cells [118]. Gene expression of tendon cells derived from embryonic stem cells was not altered with IL-1β, possibly due to lower expression of IL-1 receptors and increased expression of IL-1 decoy receptors. This model suggests that tendon cells derived from embryonic stem cells retain their reduced response to inflammatory cytokines (e.g., IL-1β). Additionally, blocking IL-1 receptors may limit adult tendon pathology [127]. Impacts of IL-1β were also explored in isolated E17 and P7 mouse tendon cells [116]. When directly compared to E15 cells, P7 cells treated with IL- 1β for 24 h upregulated inflammatory mediators, specifically IL-6, tumor necrosis factor (TNF) α, COX2, MMP-3 and MMP-13 [116]. Together, these in vitro models showed that postnatal and adult tendon cells have an inflammatory response to IL-1β, which may contribute to poor postnatal tendon healing and scar formation, and are intrinsically different from embryonic cells. Improved understanding of the pathways regulating scarless healing in embryonic and neonatal tendons may advance adult tendon healing strategies.

### Knockout and overexpression models

Animal models have been developed to explore impacts of specific proteins on tendon injury and healing. Tenomodulin knockout mice and wild-type controls underwent Achilles tendon transection and surgical repair [119]. Col I, tenascin-C, thrombospondin 2, and TGFβ1 were downregulated in tenomodulin knockouts, but scleraxis was upregulated, along with chondrogenic genes, COMP and proteoglycan 4. Compared to wild-type controls, scar tissue in tenomodulin knockout mice was more disorganized and had increased adipocyte and blood vessel accumulation, apoptosis, and reduced tendon cell proliferation. These findings suggest that tenomodulin may be an important factor in regulating adult tendon healing.

Mohawk may be involved in tendon cell responses to loading and healing. Cells isolated from patellar tendons of 3 week old mohawk knockout rats and subjected to 4% cyclic tensile loading for 6 h in vitro had increased chondrogenic gene expression, compared to control cells from mohawk ^+^/^+^ animals [47]. Cyclic loading of tendon cells from mohawk ^+^/^+^ rats increased expression of the tenogenic genes, mohawk, and Col I and III [47], suggesting that mohawk plays a role in mechanoregulation. Partial transections of rat Achilles tendons repaired with scaffolds seeded with bone marrow-derived MSCs had increased expression of mohawk, Col I, tenascin C, and tenomodulin, compared to defects repaired with a cell-free scaffold, suggesting that mohawk is expressed in MSCs during repair [32]. Another in vivo injury model repaired full transections of adult mouse Achilles tendon with cell sheets composed of mohawk overexpressing mouse MSCs [31]. After 4 weeks of healing, tendons repaired with mohawk-overexpressing cell sheets had collagen fibrils with increased diameter and a visible crimp pattern, and increased stiffness, elastic modulus, maximum force and stress, compared to repairs using cell sheets that contained wild type MSCs [31]. Overall, mohawk expression appeared to enhance tendon healing. As mohawk expression is suppressed in human tendinopathy [144], interventions regulating mohawk expression may have potential for preventing and treating tendon injuries.

GDF-5 has also been explored in tendon healing. GDF-5-null 8 week old mice with an induced Achilles tendon injury lagged 5 to 9 days behind wild-type mice in attaining peak values for normalized DNA, GAG, and hydroxyproline content [120]. Compared to wild-type controls, tendons of GDF-5-null mice had increased collagen fibril disorganization and adipose cells, and reduced collagen fibril area fraction and orientation [120]. However, despite the initial delay, at 12 weeks both groups had similar structural properties, suggesting that other factors may be able to promote healing in the absence of GDF-5 [120]. Redundancy and overlap in many signaling pathways are a persistent challenge in understanding the biochemical factors in tendon injury, but GDF-5 may regulate early tendon healing.

Decorin and biglycan, small leucine rich proteoglycans, have been implicated in the mechanical properties and aging of tendon [171, 172], and have been investigated in the response to injury. Biglycan-null and decorin-null mice were subjected to a full thickness, partial width patellar tendon injury at P120 [121]. At 3- and 6-weekspost-injury, all injured tendons contained smaller diameter collagen fibrils, compared to uninjured controls, but biglycan-null tendons had fewer of the largest diameter fibrils. Furthermore, decorin-null and biglycan-null tendons had decreased cell density, and altered cell shape and collagen alignment following injury [121]. Overall, this model suggested that early healing is influenced by biglycan, while healing 6 weeks post-injury is impaired in the absence of decorin. In a follow-up study, the same injury model was evaluated in P270 decorin-null and biglycan-null mice to determine impacts of age on patellar tendon healing [122]. At 3 weeks post-injury, tendon healing was delayed in both biglycan-null and decorin-null mice, compared to wild-type control tendons that had a higher dynamic modulus [122]. These findings contrast with injury at P120, where biglycan-null mice were deficient in healing at 3 weeks post-injury, while decorin-null mice healed more poorly at 6 weeks post-injury [121]. Together, these models show that decorin and biglycan impact tendon healing differently depending on age, which highlights age as an important consideration in injury models.

### Models of chronic injury

The models discussed above have featured mainly acute injuries. Chronic tendon injuries are challenging to develop in models due to the multitude of contributing and unknown factors and the long timescales associated with pathologies. Nevertheless, chronic injury models have been developed by altering mechanical loading or biochemical factors. An in vivo model used 10-week-oldscleraxis-GFP mice to compare the chronic loss and gradual return of mechanical loading through botulinum toxin A (Botox), to an acute loss of mechanical loading (transection) [123]. At 3 days after Achilles tendon transection, 70% fewer tendon cells remained in the injury site (cell death was mainly via apoptosis), and scleraxis expression was irreversibly lost in most remaining cells [123]. However, when tensile loading was reversibly lost (via Botox) and gradually restored, there was still apoptosis, but a larger proportion of remaining tendon cells expressed scleraxis [123]. A TGFβ1 receptor inhibitor (SD208) prevented massive tendon cell death in transected tendons, suggesting loss of tension by transection resulted in TGFβ1 signaling that induced apoptosis. In the same study, when Achilles tendon cells were isolated and cultured in vitro, scleraxis expression decreased, but fluid flow-induced shear force restored scleraxis expression [123]. These in vivo and in vitro models showed that both chronic and acute loss of loading impact scleraxis expression and cell viability.

Another animal model evaluated healing of chronic and acute rotator cuff injuries [124]. Rat supraspinatus and infraspinatus tendons were transected and then surgically repaired after a delay of 8 or 16 weeks for the chronic case, or repaired immediately for the acute case. Compared to tendons injured and repaired immediately, tendons repaired after 8 weeks showed reduced toughness, elastic modulus, and stiffness when assessed at 4 weeks after reparative surgery [124]. Scar tissue formation and tendon retraction made surgery difficult in the delayed repair cases. Rats were not immobilized following injury, which may have led to larger tears and worse outcomes in the chronic injury groups [124]. This model showed the direct impact of a chronic versus an acute injury.

TGFβ1 is found in injured tendon, and may initiate inflammation via the hypoxia-inducible factor (HIF)1α pathway [173, 174]. Another chronic tendinopathy model was developed by injecting human TGFβ1 in adult mouse Achilles tendons [125]. This TGFβ1-injection model of tendinopathy also explored the role of glucose metabolism in tendon injury in both wild type and Adamts5^−^/^−^ (TS5KO) knockout mice [125]. The production of lactate from glucose breakdown during hypoxia or normoxia is implicated in chronic tendinopathy and may be a metabolic marker of tendon disease [175]. TS5KO mice have reduced or absent osteoarthritis following surgical joint injuries, since they lack the inflammatory aggrecanase ADAMTS5, and have a diminished response to inflammatory mediators such as TGFβ1. TGFβ1 injections in Achilles tendons in vivo and ex vivo upregulated several HIF1α, angiogenesis, and glycolytic metabolism associated genes in wild-type mice, but not in TS5KO mice. TGFβ1 injections activated the Warburg pathway, which generates lactate from glucose under normoxia rather than just hypoxia, inhibits mitochondrial energy production, and contributes to tendinopathy [125]. Taken together, this model showed that TGFβ1-induced glycolytic reprogramming contributes to pathogenic responses in tendons. Therapies aimed at blocking this metabolic shift may have clinical potential.

#### Computational models of tendon pathology

Computational models of tendon pathology have been used for assessing the causes, onset, and progression of tendon damage at both the cell and bulk tissue levels. Computational models provide insights that are otherwise difficult to obtain in an experimental setting, such as stress distributions in tendon. To understand stress distributions associated with injury, 2D finite element analysis (FEA) has been used [176, 136]. FEA was used to model stress concentrations in partial-thickness defects in the rotator cuff, and highlighted the importance of limiting mechanical loading to prevent worsening of partial tears [176]. Another 2D FEA model predicted locations of increased strain and isolated tendon fascicle damage in “jumpers knee,” a common patellar tendon injury with previously unknown etiology [136]. Evaluation of the model using cadaveric patella-patellartendon-tibia samples showed that the predicted loading conditions with the highest local strain induced tendon fascicle disruption in 3 of the 5 samples, at the anatomical location of reported pain [136]. This FEA model was later used to assess infrapatellar straps, a device used to reduce patellar tendon pain, and showed that strain was effectively decreased by the strap [177]. FEA models can be useful in assessing forces on tendon, and evaluating invasive and non-invasive interventions, but impacts on cell behavior cannot be easily integrated.

Injury alters the cellular, biochemical and mechanical characteristics of tendon. These changes can be challenging to express mathematically, but several tendon injury models are based on Hill equations, which are commonly used to model cellular responses, particularly secretion or degradation of molecules or ligands [178, 179]. A three-componentHill-type equation model was used to incorporate mechanical and strain-dependent proteolytic collagen fiber damage in a human Achilles-soleus tendon unit [134]. The model predicted that proteolytic damage would result in collagen fiber shortening, while mechanical damage would result in overall fiber lengthening [134], thus showing that collagen fiber damage and resulting length after healing is modulated differently in overuse versus inflammation injuries. Predicting how collagen is altered by various damage and repair mechanisms will help guide treatments and prevent re-injury during rehabilitation.

**Fig. 4:**
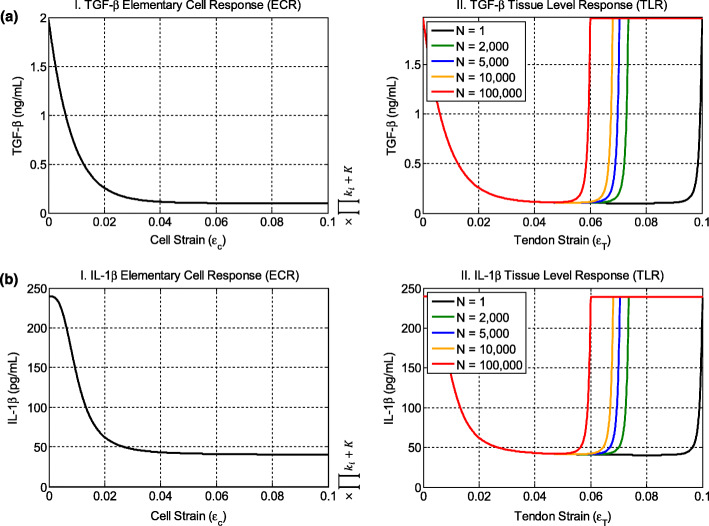
Computational modeling of cell- and tissue-level secretion profiles for inflammatory mediators in response to strain. TGFβ1 (**a**) and IL-1β (**b**) profiles were predicted by a Hill-equation model for individual cells, the elementary cell response (ECR), and for cells in the whole tendon, tissue-level response (TLR). In the TLR, the secretion profile is U-shaped, as both low and high strain lead to a simulated “unloading” response of the tissue. Low tendon strain is “underuse” and high tendon strain leads to collagen fiber rupture and unloading of the cells. Figure reprinted with permission by Springer-Verlag Berlin Heidelberg from Mehdizadeh et al. 2017 [133]

ECM and inflammatory protein secretion by tendon cells has been modeled using a modified Hill equation [133]. Secretion profiles of IL-1β, MMP-1, Col I, and TGFβ1 were predicted in response to tensile strain magnitude. A low (4%) and high (10%) strain applied to the tendon model both resulted in a damage response. A low tissue strain resulted in cell-level strain that was too low to elicit a cell response (e.g., underloading), and at high tissue strain (e.g., overloading), the collagen fibers ruptured and could no longer transfer localized strain to the cells, leading to ECM protein secretion profiles similar to the low strain condition [133]. Therefore, both low and high intensity loading increased inflammatory markers IL-1β and MMP-1, and decreased Col I. Based on these predicted cell expression profiles, quantitative thresholds for tendon mechanical under-stimulation (e.g., underuse) or overstimulation (e.g., overuse) were developed (Fig. 4). Predicting tendon cell responses to various mechanical loads can guide therapies for promoting tendon homeostasis.

A regression model was developed to assess the mechanical properties of developing and healing Achilles tendons in mice with injuries induced at P7 or P21, and with 3 or 10 days of healing [179]. Proteoglycans were found to predict tendon elastic modulus during early healing, but not during later healing or during normal development (early or late) [135]. While multiple independent parameters predicted stress relaxation during normal development, only biglycan and collagen fibril diameter predicted the percent relaxation in the tendon during early healing [135]. Using regression analysis, it may be possible to predict outcomes based on specific measurable factors. In a different study, healing of a supraspinatus tendon injury with mechanical loading was evaluated using an agent-based computational model. The model predicted that collagen content would increase steadily with increasing load, whereas collagen alignment would peak at an intermediate strain, and then decline at higher strain [137]. Peak collagen alignment occurred at a slightly lower strain level than peak collagen content. Notably, collagen deposition peaked after the damage threshold, suggesting that sub-damage threshold loading may be used therapeutically to optimize both collagen deposition and alignment. Tendon mechanical function after injury is largely determined by its underlying collagen structure, which may depend on the degree of mechanical loading experienced during healing, therefore understanding strain magnitude-dependent mechanisms of collagen remodeling is needed for developing therapies [137].

Various animal models of Achilles tendon healing have produced conflicting results, possibly due to differences in mechanical loading during healing. A multiscale computational model of rat Achilles tendon healing was developed to address this experimental variability and incorporate the loading environment to study impacts on cell behavior, collagen deposition, and scar formation [138]. The model generated a single set of cellular response curves that were able to explain observations of tendon behavior in several experimental studies with otherwise differing results [138]. The model successfully predicted cell-level behaviors from tissue-level strains, highlighting disparities in strains between cells and bulk tissues as a factor contributing to contradictory experimental results*,* and offering the possibility of reconciling these variances.

Empirical models have been developed to assess the progression of mechanical damage with injury and aging [180, 139]. In these models, damaged tendons are considered to be experiencing a lower strain than what is actually applied [180]. Based on this concept, mouse patellar tendons were evaluated as a function of age at P150, P300, and P570, and compared to P120 patellar tendons at 3- and 6-weeks after a full thickness, partial width injury [139]. Tendons were mechanically evaluated with a 10-cycle frequency sweep of 0.125% amplitude sinusoidal strain at frequencies of 0.01, 0.1, 1, 5, and 10 Hz superimposed onto a baseline offset strain (4, 6, or 8%). The equilibrium stress, dynamic modulus, and loss tangent were measured at each frequency and strain level, and an empirical model was used to develop a single damage parameter for each tendon group. The damage parameter was able to predict dynamic modulus and loss tangent for each tendon across frequency (0.01–10 Hz) and strain (4–8%). This model showed that the effects of aging and injury on patellar tendon mechanical properties could be described by the same damage model [139]. A similar strain-based empirical damage model was developed to examine impacts of decorin and biglycan knockout on Achilles tendons of P150, P300, and P570 mice [140]. The empirical damage model predicted the changes in dynamic modulus that resulted from the null phenotypes, and identified a correlation between measured and predicted dynamic modulus based on genotypes and ages [140]. Overall, these models are useful tools for understanding and predicting tendon mechanics with age, genotype, and injury.

Alongside their considerable potential for providing insight into tendon injury and healing, computational models have inherent limitations. Most simplify multiple parameters of tendon responses to load and damage. Baseline values for tendon material properties, such as elastic modulus, are obtained from previous studies, but elastic modulus varies based on tendon and species [181]. Finally, as not all proteins involved in injury and healing are known, all models necessarily exclude some cellular responses to tendon injury. Nevertheless, computational models are proving useful as research tools and predictors of tendon responses to many physiological conditions. They will undoubtedly improve further as experimental studies continue to uncover mechanisms that regulate tendon development, injury and healing.

## Conclusions and future directions

The high cell density, low collagen content, growth factors, and mechanical environment of embryonic tendon development have been incorporated into engineered model systems. Embryonic tendon becomes mechanically stronger, but differentiation and tissue formation continue postnatally, before maturation into adult tendon. Assessing the changes that postnatal tendons undergo through in vitro models remains an ongoing challenge. Furthermore, many biochemical and mechanical cues inevitably originate from surrounding tissues. The impacts of concurrent adjacent tissue formation (muscle and bone) on tenogenesis need to be explored, as simulating these tissues in vitro may facilitate more realistic tendon models. Few multi-tissue developmental models exist, but one study showed that 3D in vitro skeletal muscle-tendon constructs developed ultrastructural characteristics resembling in vivo muscle-tendon interfaces, when skeletal muscle constructs where co-cultured with self-organizing tendon constructs and explanted fetal rat tail tendon [182]. Such constructs can be supplemented with biochemical or mechanical factors to better mimic the developmental process. Furthermore, examining development of the musculoskeletal system as a whole will aid in understanding how tendon formation is regulated in coordination with adjacent tissues including muscle and bone.

An additional challenge with developmental models is that recreating the spatiotemporal sequence of embryonic or postnatal biochemical signaling alone may be inadequate for developing functional tissue [183]. Several models examine specific tenogenic factors in isolation, an understandable limitation given the complexity of tendon development. Future models will need to assess the interplay between cell-level cues, mechanical loading, development of mechanical properties, and the biochemical factors involved in tendon formation.

In vivo*,* in vitro*,* ex vivo, and computational models have explored the impacts of mechanical loading and various biochemical factors on adult tendon injuries and healing. Few models have investigated human derived cells or isolated human tissues, mainly due to the understandable challenge of procuring tissues and working with human subjects. Recent studies in other tissue systems have developed humanized models (e.g. decellularized animal tissues seeded with human cells or humanized animal models) [184, 185], but this has not been explored as thoroughly in tendon. Advancing models of chronic tendon injuries are needed for exploring the factors that regulate tendon pathologies in human tissues and cells. Taken together, the developmental and injury models reviewed here have greatly improved our understanding of the numerous cellular, biochemical and mechanical factors that regulate tendon formation and health. Tendon models will ultimately improve clinical outcomes by offering novel insights into the mechanisms of how tendons develop and how they respond to injury and treatment.

## Data Availability

Not applicable.

## Abbreviations

Adamts5: A disintegrin and metalloproteinase with thrombospondin motifs 5
BMP: Bone morphogenetic protein
Col: Collagen
COMP: Cartilage oligomeric matrix protein
COX2: Cyclooxygenase 2
CTGF: Connective tissue growth factor
D: Dimensional
E: Embryonic day
ECM: Extracellular matrix
EGR: Early growth response
FEA: Finite element analysis
FGF: Fibroblast growth factor
GAG: Glycosaminoglycan
GDF: Growth and differentiation factor
GFP: Green fluorescent protein
HIF: Hypoxia-inducible factor
IGF: Insulin-like growth factor
IL: Interleukin
LOX: Lysyl oxidase
MMP: Matrix metalloproteinase
MSCs: Mesenchymal stem cells
MT: Membrane type
P: Postnatal day
PGE: Prostaglandin
RGD: Arginyl-glycyl-aspartic acid
SEM: Scanning electron microscopy
siRNA: small interfering RNA
TGF: Transforming growth factor
TGFR: Transforming growth factor receptor
TIMP: tissue inhibitors of metalloproteinases
TNF: Tumor necrosis factor
